# Comparison of Noninvasive Imagery Methods to Observe Healthy and Degenerated Olfactory Epithelium in Mice for the Early Diagnosis of Neurodegenerative Diseases

**DOI:** 10.3389/fnana.2020.00034

**Published:** 2020-07-14

**Authors:** Adeline Etievant, Julie Monnin, Thomas Lihoreau, Brahim Tamadazte, Patrick Rougeot, Eloi Magnin, Laurent Tavernier, Lionel Pazart, Emmanuel Haffen

**Affiliations:** ^1^Laboratoire de Neurosciences Intégratives et Cliniques, Université Bourgogne-Franche-Comté, Université de Franche-Comté, Besançon, France; ^2^CHU Besançon, INSERM, CIC 1431, Centre d'Investigation Clinique, Besançon, France; ^3^FEMTO-ST, Dép. AS2M, CNRS, Université Bourgogne Franche-Comté, 24 rue Savary, Besançon, France; ^4^Institut des Systémes Intelligents et de Robotique, Sorbonne Université, CNRS, UMR 7222, Paris, France; ^5^Service d'oto-Rhino-Laryngologie et Chirurgie Cervico-Faciale, CHU Besançon, Université Bourgogne-Franche-Comté, Besançon, France

**Keywords:** epithelium olfactory, medical imaging, optical biopsy, neurodegenerative diseases, Alzheimer disease

## Abstract

Olfactory dysfunction could be an early and reliable indicator for the diagnosis of neurodegenerative disorders such as Alzheimer and Parkinson's diseases. In this paper, we compare the potential of different noninvasive medical imaging modalities (optical coherence tomography, confocal microscopy, and fluorescence endomicroscopy) to distinguish how the olfactory epithelium, both at the cellular and the structural levels, is altered. Investigations were carried out on three experimental groups: two pathological groups (mice models with deliberately altered olfactory epithelium and Alzheimer's disease transgenic mice models) were compared with healthy mice models. As histological staining, the three tested noninvasive imaging tools demonstrated the general tubular organization of the olfactory epithelium on healthy mice. Contrary to OCT, confocal microscopy, and endomicroscopy allowed visualizing the inner structure of olfactory epithelium as well as its morphological or functional changes on pathological models, alterations classically observed with histological assessment. The results could lead to relevant development of imaging tools for noninvasive and early diagnosis of neurodegenerative diseases through the *in situ* characterization of the olfactory epithelium.

## 1. Introduction

**Figure 1:**
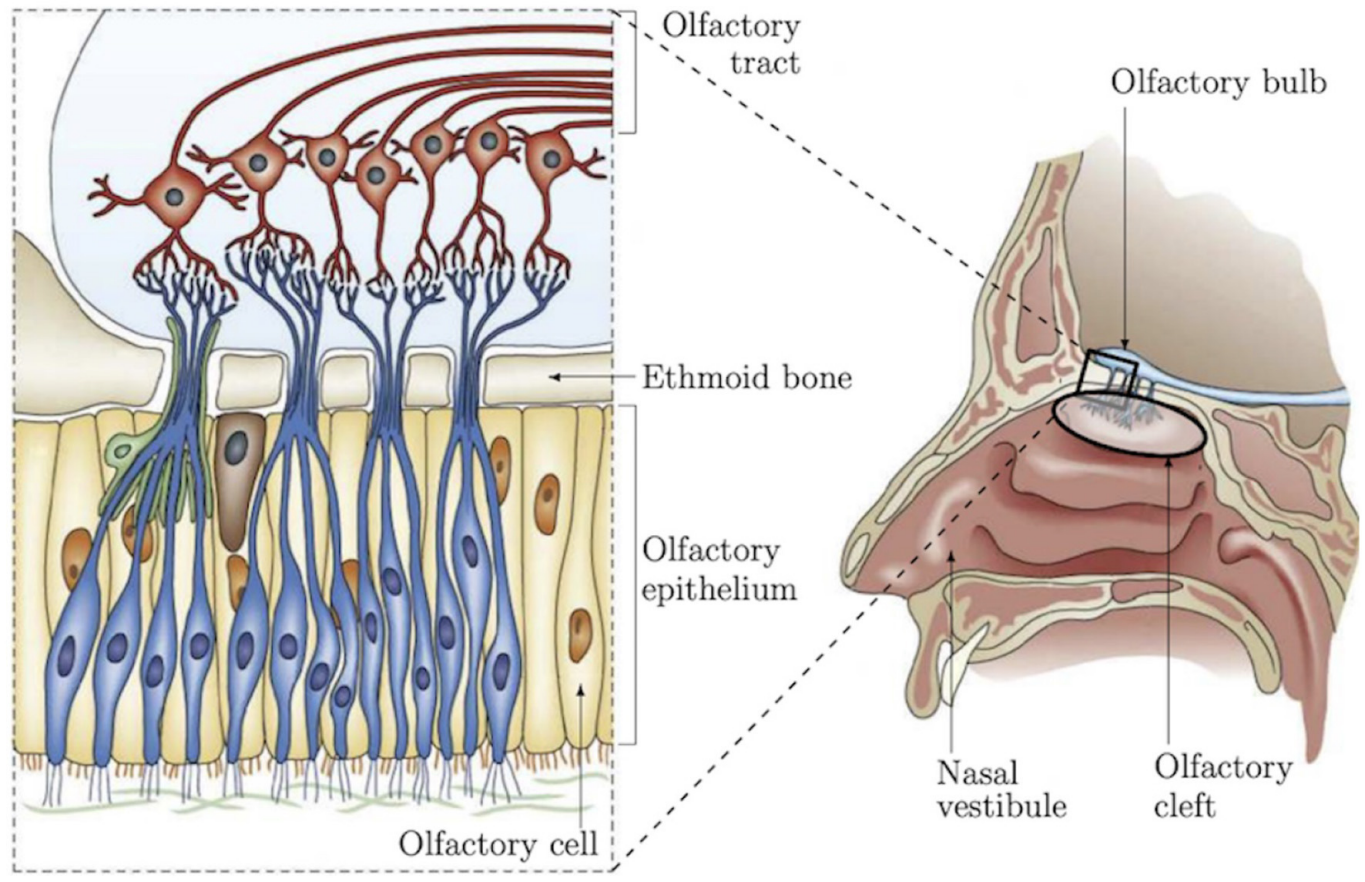
Representation of nasal anatomy, structure, and the OE shape and location (Girerd et al., 2018).

Recent studies have shown a strong correlation between impaired olfactory perception of patients and neurodegenerative conditions, such as Alzheimer's disease (AD) (Arnold et al., 1998; Wang et al., 2010; Wesson et al., 2010; Kjelvik et al., 2014), Parkinson's disease (Berg, 2008; Doty, 2012), frontotemporal dementia (McLaughlin and Westervelt, 2008; Alves et al., 2014), and Huntington's disease (Lazic et al., 2007; Barresi et al., 2012). These works lead to consider olfactory dysfunction as an early marker of neurodegenerative conditions and as a relevant indicator for early-stage diagnosis of such diseases. For instance, in AD, odor detection, discrimination and identification are affected earlier than cognitive performances as demonstrated in several studies on patients (Talamo et al., 1991; Arnold et al., 1998; Wang et al., 2010), as well as on different animal models, in particular mice (Sohrabi et al., 2012; Alvarado-Martínez et al., 2013; Wu et al., 2013). These functional olfactory alterations are probably due to early Amyloid-β peptide deposits in the olfactory epithelium (OE) leading to cellular apoptosis and a decrease of dendritic spine densities (Yao et al., 2017). These studies have identified a need to investigate, in a more advanced manner, the area of the nasal cavity which concentrates part of the olfactory functionalities in order to establish reliable biomarkers of AD. This will serve to both improve diagnosis and to surrogate markers of efficacy during clinical trials (Quinn, 2013). Olfactory epithelium is a pseudo-stratified neuroepithelium covering 10% of the nasal cavity and responsible of odor detection. It is characterized by three main cell types that can be clearly identified: the olfactory sensory neurons constituting the receptor cells for trapping odor molecules, the supporting cells and the basal stem cells that continuously regenerate olfactory neurons (Holbrook et al., 1995; Barrios et al., 2014). Reaching the OE for *in vivo* characterization and monitoring of the neural organization (Figure 1) is still an open scientific and clinical challenge because of its location and access pathway as demonstrated in our recent work (Girerd et al., 2018). To the best of our knowledge, no conventional instrument can be used to non-invasively reach this area. To overcome this problem, we are developing a microrobotic solution based on a concentric tube robot mechanism (flexible robotic endonasal system), which embeds the optical characterization tool such as miniature Optical Coherence Tomography (OCT), confocal, or endomicroscopy probe. This work is investigated within the translational and multi-disciplinary NEMRO project^1^ that aims at identifying neuropathological changes and early signs of degeneration within the human olfactory tissue for earlier diagnosis of neurodegenerative diseases. More precisely, the work carried out consisted in developing a nasal endoscopic system based on the use of flexible continuum robot of <2 mm of diameter able to navigate without collision within the nasal slots. The endoscopic system can be equipped, thanks to its inner free channel, with a fiber-based imaging probe (i.e., OCT, confocal, or endomicroscopy) for *in situ* characterization of the OE. Pending the design of this new system, we have implemented a series of experiments to the ability of the imaging tools to: (i) distinguish the structural shape of the OE on healthy mice by comparing the results with those using conventional histological assessment, and (ii) identify morphological alterations and early signs of degeneration using pathological mice models (ZnSO4 lesion, APPswe/PSEN1E9 mice model of AD).

Confocal microscopy, OCT, and endomicroscopy are widely studied in both research investigations and clinical purposes, especially in ophthalmology and dermatology. The images produced by these imaging systems are also known as optical biopsies able to visualize biological tissues both in depth and at micrometer resolution while being non-invasive. For instance, OCT has demonstrated the ability to investigate cytoarchitecture in the brain (Ibne Mokbul, 2017) and to observe, among others, human nasal epithelium (Mahmood et al., 2006; Oltmanns et al., 2016). Confocal microscopy, a less recent technology compared to the other two, has become an interesting investigation technique in medicine (Fine et al., 1988; Hofmann-Wellenhof et al., 2012). Concerning the endomicroscopy such as the CellVizio technology, it is more recent and has proven real benefit for *in vivo* diagnosis of some diseases, namely for GI tract applications (De Palma, 2009; Mielke et al., 2015).

The experimental scenario carried out in this paper consisted in studying the potential of each of the selected imaging modalities to observe alterations (at the structural or cellular levels) that are involved within the OE tissues. To do this, two groups of mice were used: (i) mice received a bilateral *ZnSO*_4_ irrigation of the nasal cavity to induce morphological alterations of the OE (Ducray et al., 2002; McBride et al., 2003; Bon et al., 2005), and (ii) double transgenic APPswe/PSEN1E9 mice (Jackson Laboratory, USA)^2^. They are mouse model of AD whose mutations targeting Amyloid precursor protein and presenilin 1 genes (APP/PS1) are associated with early-onset of Amyloid-β peptide within the OE and the brain resulting in learning and memory deficits (Wu et al., 2013; Yao et al., 2016).

The preliminary conclusions from these experiments show that OCT allowed visualization of the general structural aspect i.e., turbinates of the OE tissues, as well as the overall disorganization of the olfactory tissue induced by ZnSO4 irrigation. However, due to the limited spatial resolution of the OCT system, this imagery tool does not allow observation at a cellular level, contrary to confocal microscopy and endomicroscopy, the pseudo-stratified structure of the OE. Indeed, confocal microscopy and endomicroscopy allowed visualizing, similarly to the histological assessment, the inner and pseudo-stratified structure of OE, i.e., cell bodies, axons, and the different cell layers of the epithelium. Otherwise, morphological changes (i.e., disorganization and reduction of the thickness of the different layers that form the OE) after ZnSO4 treatment that are traditionally observed with histological procedure were well-observed by the three imaging systems. Concerning the visualization of possible Amyloid-β peptides occurring within the tissues sampled from APPswe/PSEN1E9 mice, only the endomicroscopy device (fluorescence confocal microscopy) pointed out possible connected fluorescent dots within the OE.

## 2. Materials and Methods

### 2.1. Animal Models

Swiss female mice (Janvier Labs, FR)^3^ and APPswe/PSEN1E9 mice aged 3–4 months were maintained under both standard and controlled laboratory conditions (12:12 h under light/dark cycle) with food and water available *ad libitum*. All animal experiments comply with the ARRIVE (Animal Research: Reporting of *in vivo* Experiments^4^) guidelines and are carried out in accordance with the European Directive 2010/63/EU^5^ for the care and the use of living animals for laboratory experiments.

### 2.2. *ZnSO*_4_ Lesion

To identify the ability of the studied optical imaging tools to visualize large deterioration incurred within the OE tissues, mice received a bilateral intranasal application of *ZnSO*_4_ solution (Sigma Aldrich, FR)^6^ under general anesthesia (isoflurane). Intranasal infusion of *ZnSO*_4_ is one of the most commonly used methods to induce a massive destruction of mature olfactory neurons and decrease odor sensitivity a few days after *ZnSO*_4_ perfusion (Ducray et al., 2002). Mice were placed on their back, and each nostril was injected with 8 μl of a sterile 10% *ZnSO*_4_ solution in 0.9% sodium chloride. Immediately after *ZnSO*_4_ irrigation, mice were held with their head down for several seconds to minimize spread of the solution to the oral cavity. Since regeneration of the OE typically occurs within 7 days after *ZnSO*_4_ application, mice were perfused 4 days after the intranasal application to keep them in condition of massive alterations (McBride et al., 2003).

### 2.3. Tissues Preparation

Mice were transcardially perfused with 4% paraformaldehyde in phosphate-buffered saline (PFA in PBS) to fix tissues. The OE tissues were then removed, post-fixed overnight in 4% paraformaldehyde and cryoprotected with a 15% sucrose solution for 24 h. The tissue samples were either embedded in Tissue Tek for histological experiments or kept in PBS for a few hours before observation with the OCT and the confocal microscopy imaging tools.

### 2.4. Histology and Immunohistochemistry

Frozen coronal and sagittal sections of 10 μm were obtained using a cryostat, mounted onto clean, subbed slides, and stored at −20°C until processing. Three different protocols were carried out as described above:

- To visualize the internal structure of OE, tissue sections were rehydrated and stained with haematoxylin-eosin during 2 min. Sections were then dehydrated, and cover-slipped with Canada balsam (Carl Roth).
- Immunohistochemistry was performed in three APPswe/PSEN1E9 mice in order to visualize Amyloid-β aggregates within their OE. After rinsing in PBS-Triton (PBS-T) 0.3%, sections were exposed to polyclonal rabbit anti-Amyloid-β primary antibody (1:100; ab2539, Abcam) in milk solution (PBS-T, 1% BSA, 10% lactoprotein) for 24 h at 4°C. After several washings, sections were exposed 2 h at room temperature to either the fluorescent goat anti-rabbit IgG (1:1,000, Alexa Fluor 488, Invitrogen) or the biotinylated secondary horse anti-rabbit IgG (1:500, Vector Laboratories) in PBS. After the incubation in biotinylated secondary antibody, OE slices were exposed to an avidin horseradish peroxidase complex (ABC Elite kit, Vector Laboratories) for 1h at room temperature. The peroxidase complex was visualized after a 10 min exposure to a chromogen solution containing 0.04% 3.3' diaminobenzidine tetrahydrochloride (DAB, Sigma Aldrich) with 0.006% hydrogen peroxide in PBS. Sections were then rinsed in PBS, stained with toluidine blue for 30 s and finally dehydrated and cover-slipped with Canada balsam (Roth).
- Images were produced from an Olympus microscope B×51 set up with a ×20, ×40, or ×60 objectives equipped with an Olympus DP50 camera (Axio Imager Zeiss). To observe Amyloid-β aggregates within the entire OE using a CellVizio imaging device (Mauna Kea Technologies^7^ Paris, FR), we adapted the immunohistochemistry protocol, normally performed on brain slices, on the whole OE tissue. OE of two APPswe/PSEN1E9 mice were treated with a PBS-T 0.3% solution during 20 min to make the tissue sample permeable. Then, samples were exposed for 44 h at 4°C to polyclonal rabbit anti-Amyloid-β primary antibody (1:100, ab2539, Abcam) in milk solution (PBS-T, 1% BSA, 10% lactoprotein). After several washings, sections were exposed (4 h at room temperature) to the secondary goat anti-rabbit IgG (1:1,000, Alexa Fluor 488, Invitrogen). Olfactory epithelium tissues were then rinsed in PBS and observed with the CellVizio device.

### 2.5. Medical Imaging Devices

**Figure 2:**
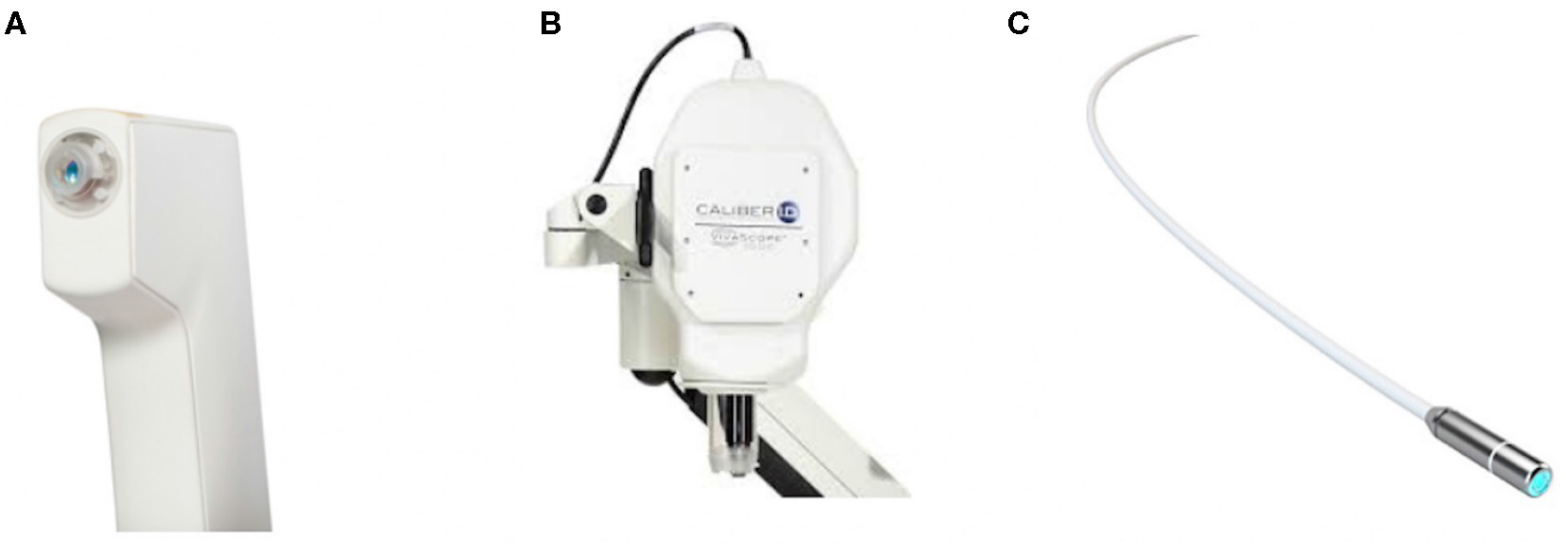
Photography of the studied imaging tools: **(A)** OCT, **(B)** confocal microscopy, and **(C)** CellVizio endomicroscopy probe.

The imaging modalities that were selected for the characterization of OE tissues are widely used in clinical applications (Figure 2). The images are commonly referred to as optical biopsies because of their ability to visualize biological tissues in depth and at micrometer resolution almost similar to a histopathological study. In addition, these images are available in a miniaturized version (or can be miniaturized) to be used *in vivo* by passing through natural orifices such as the nasal slots or through small artificial orifices.

#### 2.5.1. Optical Coherence Tomography

OCT allows observing the different tissue layers (by penetrating into the scattering medium) in aim to capture micrometer-resolution images (i.e., optical biopsies) and in nondestructive way. The Vivosight OCT device (Figure 2A) (Michelson Diagnostics^8^, UK), initially developed for clinical dermatology, was tested in this work. It uses a multi-beam swept-source frequency domain OCT (SS-OCT) equipped with a λ = 1,300 nm wavelength light source, which offers an accurate *in vivo* and in-depth characterization (up to 2 mm) of biological tissues thanks to an optical resolution of 7.5 and 5 μm laterally and axially, respectively. Three optical biopsy modes are provided by with the OCT system: optical core (1D z-signal), cross-sectional slices (2D images), and volumes. This kind of imaging tool was used in few studies on animal models which demonstrated that OCT is effective in the visualization of rat olfactory bulb (Watanabe et al., 2011) and mice hippocampus (Chong et al., 2015) or olfactory epithelium (Ueda et al., 2019).

The Vivosight OCT device was used in our work in order to visualize the different OE layers sampled from both the healthy and pathological mice. The OCT data were used to compare structurally the OE structure, shape, and thickness for both groups. The results are discussed and compared to the other imaging tools as well as the histological results reported in section 3.

#### 2.5.2. Confocal Microscopy

VivaScope 1500 (Figure 2B) (Mavig GmbH^9^, DE) is typically used to observe and evaluate biological tissues in both *in vivo* and *ex vivo* manners. Its current commercialized *in vivo* use in dermatology, allows visualization through the epidermis and dermis until the reticular layer by just putting the probe onto the skin of the patient with oil/gel interface, without any damage/pain. A laser beam (830 nm) is used and directed onto the skin area of interest and is then reflected forming (after a reconstruction phase) grayscale and real-time micrometric resolution images of the tissue. The reflectance confocal microscopy VivaScope 1500 is able to perform investigation on the tissue in the transverse plane of 5 μm of thickness with a field-of-view of 500 × 500 μm. In addition, a software is provided, which allows tuning the laser source power and then varying (with a step of 1.5 μm) the acquisition depth from the tissue surface up to 200 μm. The data can be arranged in a succession of 2D stacks. Additionally, a high-resolution actuator equips the device. It allows moving laterally the confocal probe in *x* and/or *y* axes in order to enlarge the initial field-of-view up to 8 × 8 mm (almost the entire size of the mice OE). In the literature, this imaging tool was already evaluated in few studies for *in vivo* investigations and characterization of mice corneal tissue (Chen et al., 2008; Lee et al., 2015).

The VivaScope device was slightly adapted to our study, by adding a designed sample holder, providing ergonomic adaptation for an *ex vivo* use. It allows to stabilize the sampled OE to avoid image artifacts induced by the probe motion during the scanning process. The optical biopsies are analyzed and compared in section 3.

#### 2.5.3. CellVizio Endomicroscopy

The CellVizio endomicroscopy probe (pCLE) (Figure 2C) is a standalone imaging system based on a fiber technology achieving real-time (9–12 images/second), high resolution, and *in vivo* optical subsurface tissue characterization. It allows to make more targeted biopsies and to reach more areas that were previously inaccessible for visualization. It has been demonstrated that pCLE can be used across a number of different indications: biliary strictures, lung nodules, pancreatic cysts, urology, and many other disciplines. The CellVizio incorporates a proximally-scanned fiber bundle to deliver a 488 nm wavelength laser light toward to the sample and acquire a fluorescence signal, in return. In our study, we used the Z1800 probe which incorporates a fiber bundle composed of 30,000 optical fibers, providing a lateral resolution of 3.5 μm with a field-of-view 512 × 448 pixels equivalent to 500 μm of diameter (the resulting image has the form of a disk). Furthermore, the CellVizio system provides different types of flexible probes sized from 1 to 5 mm (diameter) offering spatial resolutions of 1 to 3.5 μm, respectively able to observe tissues at different depths ranging from 0 to 70 μm depending on the probe.

Furthermore, to visualize and characterize the sampled OE at the cellular level, we prepared a biochemical solution in which the sampled OE were previously soaked. To do this, we used the Acriflavine (Sigma-Aldrich), a fluorescent agent for labeling acidic constituents, to stain nuclei (by labeling RNA molecules) of the different structures of the OE (olfactory neurons layer, connective tissue, etc.). The main particularity of the pCLE device is the possibility to emphasize the presence of Amyloid-β peptides within OE tissues. To the best of our knowledge, no such work has been reported in the literature.

## 3. Results

### 3.1. Healthy Olfactory Epithelium

**Figure 3:**
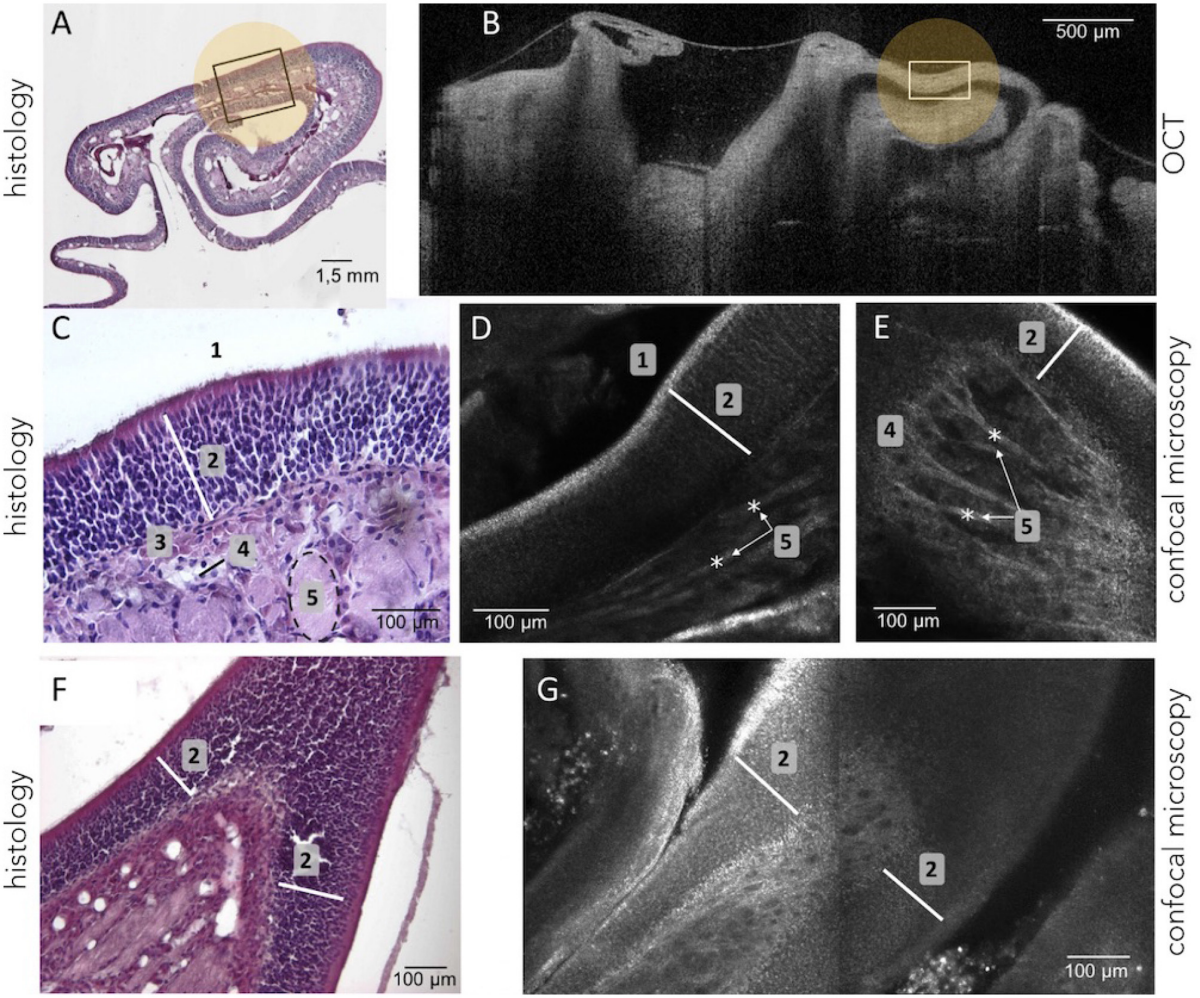
Olfactory epithelium tissues were observed using histology (remove-frozen-cut-HE stained) as shown in **(A,C,F)**. OE were also viewed using the OCT device as on **(B)**. It highlights that the same region can be identified in both histology and OCT (e.g., the area marked with a white rectangle) which showed the general organization and structure of the OE. The VivaScope confocal microscopy system **(D,E,G)**, as well as histology **(C,F)**, allowed the observation at a cellular and layered level of the OE tissues such as cilia (1), neuronal layers (2), basement membrane (3), blood vessels (4), bundles of axons (5).

As expected, in healthy mice, the main OE appeared as a pseudo-stratified structure organized in turbinates (Barrios et al., 2014) using histological assessment (Figure 3A). In Figure 3C, from the surface to the depth, we can see olfactory cilia (1-receptors), a thick layer of olfactory neurons and supporting cells (≈ 160 μm) (2), basal stem cells (3), the presence of blood vessels (4), and bundles of axons (5) within connective tissue.

The Vivosight OCT device was used for the real-time visualization of perfused OE. When, the turbinate structure of the OE could be clearly distinguished because of the different shades of gray visible in Figure 3B, the spatial resolution (≈ 5μm) did not allow to highlight its internal structural organization (i.e., olfactory neuronal layers) as shown in Figures 3A,B (rectangular boxes).

The confocal microscopy device, which provided a higher resolution compared to OCT, allowed to visualize both the general shape of the turbinates and the inner structure of the OE tissues, and to measure its thicknesses (≈ 140 μm). Confocal images allowed to identify internal structures at almost cellular scale, as demonstrated in Figures 3D,E. Indeed, the thin hyper reflecting and irregular superficial layer corresponding to cilia receptors can be seen, as well as the layers of the olfactory neurons and the axons bundles. Images produced during optical microscopic characterization (Figure 3F) and the confocal microscopy examination (Figure 3G) were substantially identical and highlight the relevant use of confocal microscopy to explore and characterize OE tissue samples. Whereas, the biochemical histology examination requires sampling, sample preparation, labeling to assess the tissue features, using confocal microscopy seems to be relevant for *in vivo* and non-invasive characterization.

### 3.2. *ZnSO*_4_-Induced Lesion of the Olfactory Epithelium

**Figure 4:**
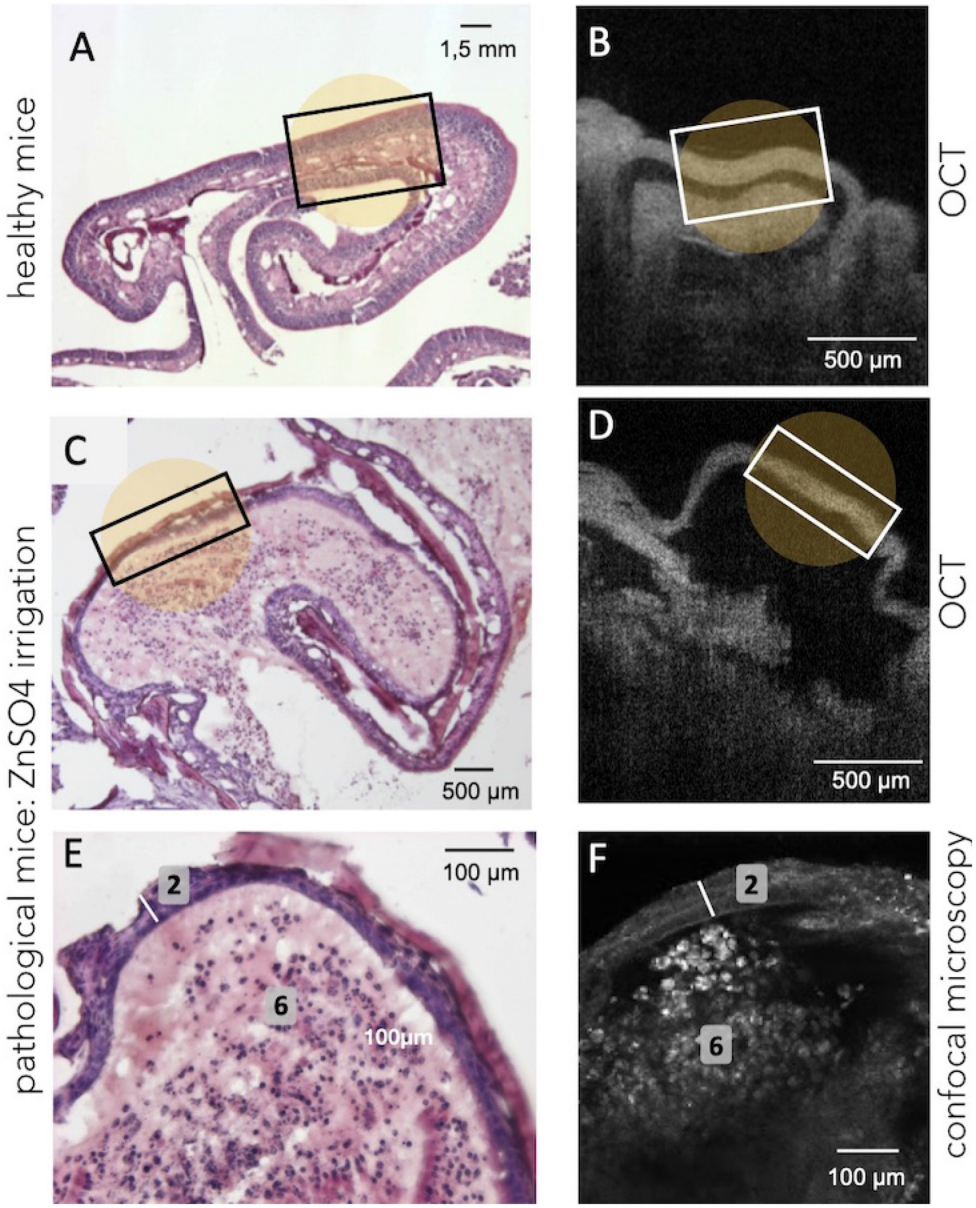
Illustration of the structural changes in OE tissue induced by bilateral *ZnSO*_4_ irrigation of the nasal cavity. **(A,B)** Show the histology and the OCT observations on healthy mice, respectively, when **(C,D)** show the tissue (at the turbinates level) after the bilateral *ZnSO*_4_ administration which resulted, for instance, in a decrease in tissue thickness. Confocal examination **(F)** offers a cellular-level observation almost similar to the histological labeling **(E)** and confirms the decreased thickness of the neuronal layer (2, white bar) and the general disorganization of the connective tissue (6, absence of blood vessels and bundles of axons) after *ZnSO*_4_ irrigation.

In order to evaluate and to compare the ability of the different optical tools to visualize major alterations within the OE, mice received an intranasal injection of *ZnSO*_4_ solution under general anesthesia (isoflurane). Intranasal administration of *ZnSO*_4_ is one of the most commonly used methods to induce a massive destruction of mature olfactory neurons and decrease odor sensitivity a few days after the treatment (Ducray et al., 2002). As demonstrated in Figure 4, an intranasal injection of *ZnSO*_4_ solution strongly injured the OE (Figures 4C,E), which appeared friable and disorganized, with a huge decrease in neuronal layer thickness of the OE (≈ 25 μm;) compared to the OE sampled from healthy mice (Figure 4A). Moreover, disorganized cell bodies seemed degraded and blood vessels and bundles of axons were no longer observed (Figure 4E).

In the same manner, OCT technique allows visualizing a structural disorganization as well as a significant reduction of the thickness of OE after an intranasal administration of the *ZnSO*_4_ solution (Figure 4D) compared to healthy mice (Figure 4B). However, it remains challenging to accurately distinguish smaller variations or damages using the OCT tool due to its limited resolution. On the contrary, confocal microscopy is more suitable to visualize morphological changes of the OE at the cellular scale: bilateral *ZnSO*_4_ irrigation of the nasal cavity damaged both at the cellular level (neuronal layers) and the general structure of the tissues (OE thickness ≈25 μm, see Figure 4F) compared to healthy OE (Figures 3A,B).

### 3.3. Mouse Model of Alzheimer Disease (APP/PS1 Mice)

**Figure 5:**
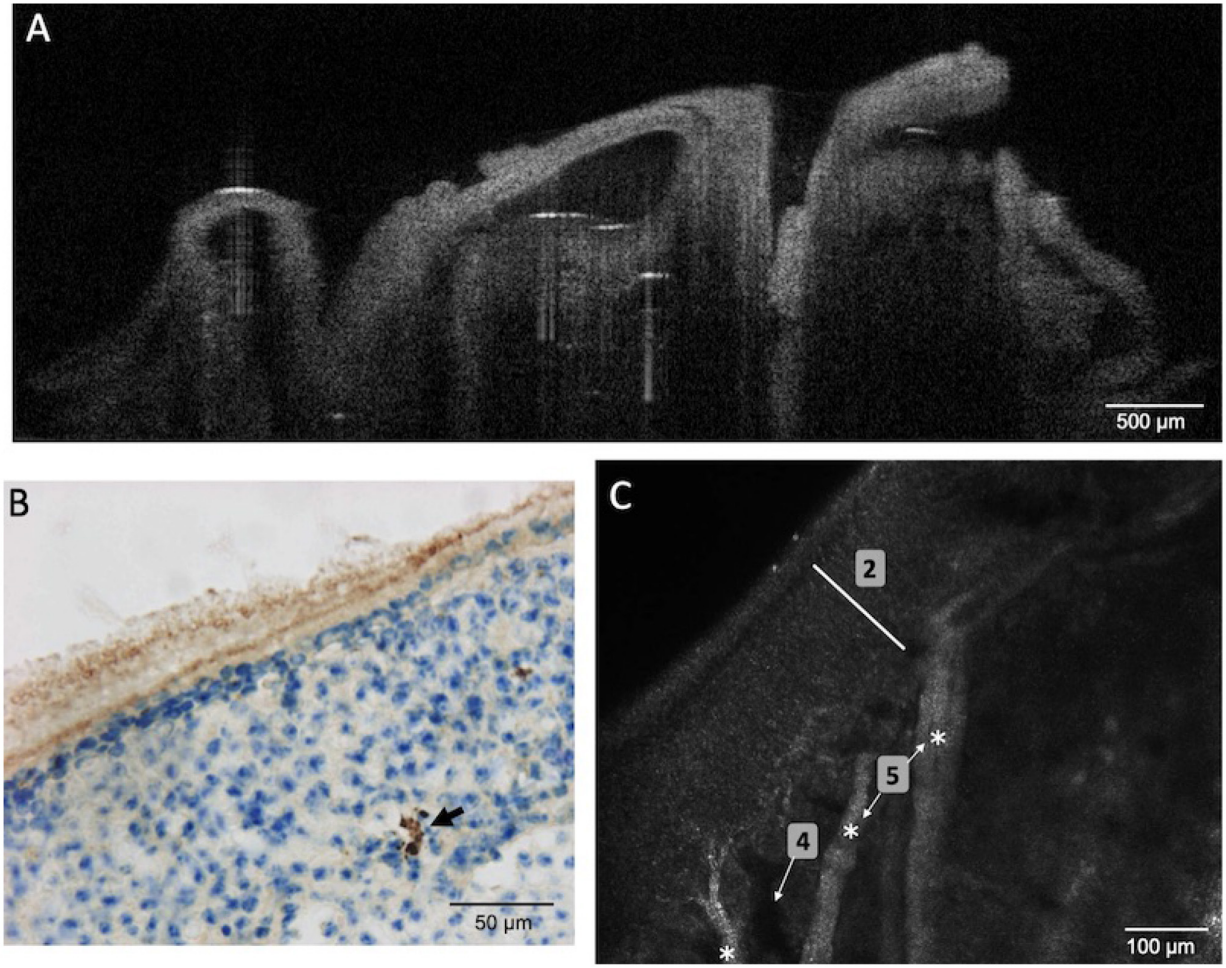
OE tissues of young APP/PS1 mice (aged 4 months) was observed using OCT **(A)**, immunohistochemistry **(B)**, or confocal microscopy **(C)**. Amyloid-β peptides were identified (black arrow) using immunohistochemistry with specific antibodies. However, OCT and confocal images failed to reveal these peptides or any alteration of the OE [2 (white bar) = neuronal layer; 4 = blood vessels; 5 (white stars) = bundles of axons].

As already established in Wu et al. (2013), immunohistochemistry allows identifying Amyloid-β peptides within the OE neuronal layer of 4 months old APP/PS1 mice model of AD (Figure 5B). As expected, OCT enables to visualize the turbinate structure and confocal microscopy enables to see the cellular organization (layer of olfactory neurons and bundles of axons), the shape, and to measure the thickness (≈ 180 μm) of the OE of APP/PS1 mice (Figure 5). However, none of these two tools give any visual clues concerning the presence of Amyloid-β peptide within the neurons layers or a decreased thickness of the OE of these mice.

**Figure 6:**
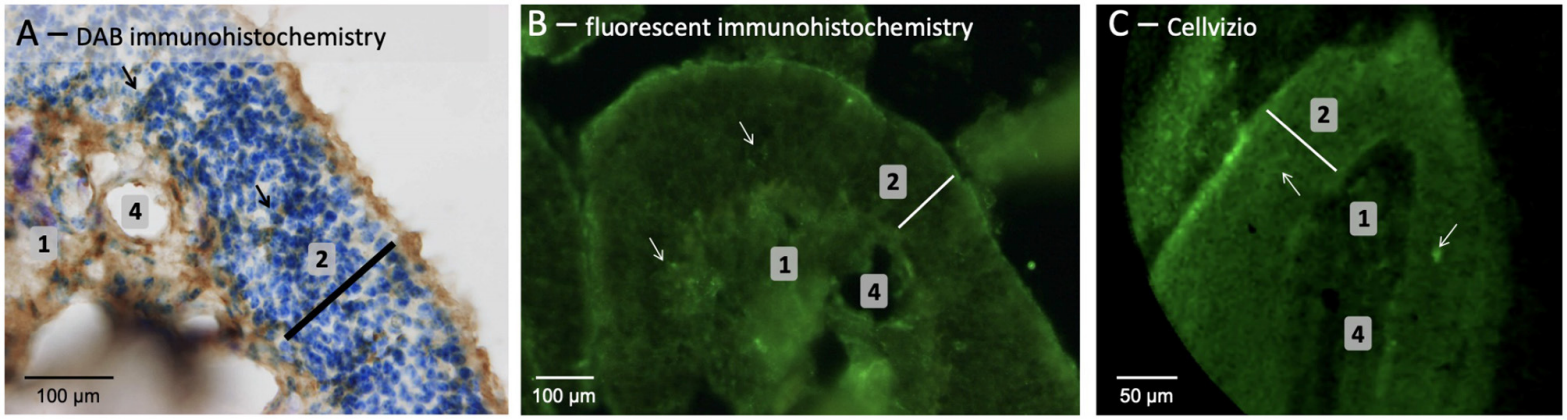
OE tissues sampled from old APP/PS1 mice (aged 18 months) was observed using classic **(A)** and fluorescent **(B)** immunohistochemistry or noninvasive CellVizio technology **(C)**. [1 = connective tissue; 2 (white bar) = neuronal layer; 4 = presumed blood vessels; arrow = presumed Amyloid-β peptide].

The 2 mm diameter standalone endomicroscopy device was used in order to evaluate the possibility to consider such system for *in vivo* investigation. The objectives are to visualize both the structural and the functional alterations of OE (i.e., potential decrease of thickness and Amyloid-β peptides). Note that CellVizio system requires the use of a fluorescence technique, which could potentially make its use on patients less trivial in comparison to OCT or confocal microscopy. However, CellVizio system has already been used in several clinical application with a well-established tissue labeling routine. Concerning our study on animal OE tissues, as depicted in Figure 6A, an immunohistochemistry followed by DAB revelation performed in OE slices of old APP/PS1 mice (18 months) highlighted that Amyloid-β peptides were organized in diffuse plaques with an irregular shape that appeared as a loose network and without a dense core when compared with 4-months-old mice.

Additionally, the fluorescent labeling of Amyloid-β peptides of the OE slices using immunohistochemistry (Figure 6B) or of whole tissue using CellVizio endomicroscopy (Figure 6C) allowed visualizing the structural organization of the tissues. By analyzing Figures 6B,C, it is possible to clearly observe the OE inner structure [i.e., olfactory neurons layer (2) laying on connective tissue (1) with altered blood vessels (4)] and allowed thickness measurement of the tissue samples (≈ 100 vs. ≈ 140 μm for histological examination). Note that observations were more challenging due to the fact that the OE seems “crumbly,” possibly because of the older mice which lead to increase the number of Amyloid-β deposits and senile plaques as demonstrated in Wu et al. (2013) and Yao et al. (2017). Presumed amyloid-β peptides could be detected if we considered the very high brightness (compared to the rest of the tissues) spots (white arrows in Figure 6C), possibly due to the presence of anti-Amyloide-β primary antibody revealed with fluorescent secondary antibody.

## 4. Discussions and Conclusion

The comparison between conventional histology and noninvasive imaging techniques, such as OCT and confocal microscopy, showed that OCT technique allowed for the macroscopic visualization of the nasal cavity content. For instance, the turbinates as well as the overall OE can be distinguished using an OCT device, while observing the different cell layers within the OE is still more challenging. In addition, OCT technique has demonstrated the possibility to observe major impairments of OE (e.g., epithelium thickness) after *ZnSO*_4_ administration, although no specific results were obtained on aged APP/PS1 mice. On the contrary, confocal microscopy allowed the observation of the macroscopic and microscopic organization of the OE. In fact, it is possible to distinguish cell bodies, axons and the different cell layers within the OE tissues. Furthermore, the major disorganization and destruction observed within OE tissues induced by the *ZnSO*_4_ treatment was clearly identified, but no specific abnormality was observed in APP/PS1 mice.

The proposed study underlined that the OCT technique, though able to characterize macroscopic aspect of the OE, is still limited to observe changes at the cellular scale, especially in term of spatial resolution. When a recent study reinforces this conclusion (Ueda et al., 2019), some reported works have demonstrated, using more advanced OCT devices, that this technique allowed observing more structural details. For instance, in Watanabe et al. (2011), authors highlighted layered organization of the rat olfactory epithelium. Recently, new generation of OCT systems, such as polarization sensitive OCT (PS-OCT) or microcontrast OCT (MC-OCT) are expected to offer a micrometer resolution optical investigation (i.e., 5–7 times better than our OCT system) as demonstrated in recent works dealing with the visualization of nerve fiber pathways in a rat's brain (Wang et al., 2014a,b).

**Figure 7:**
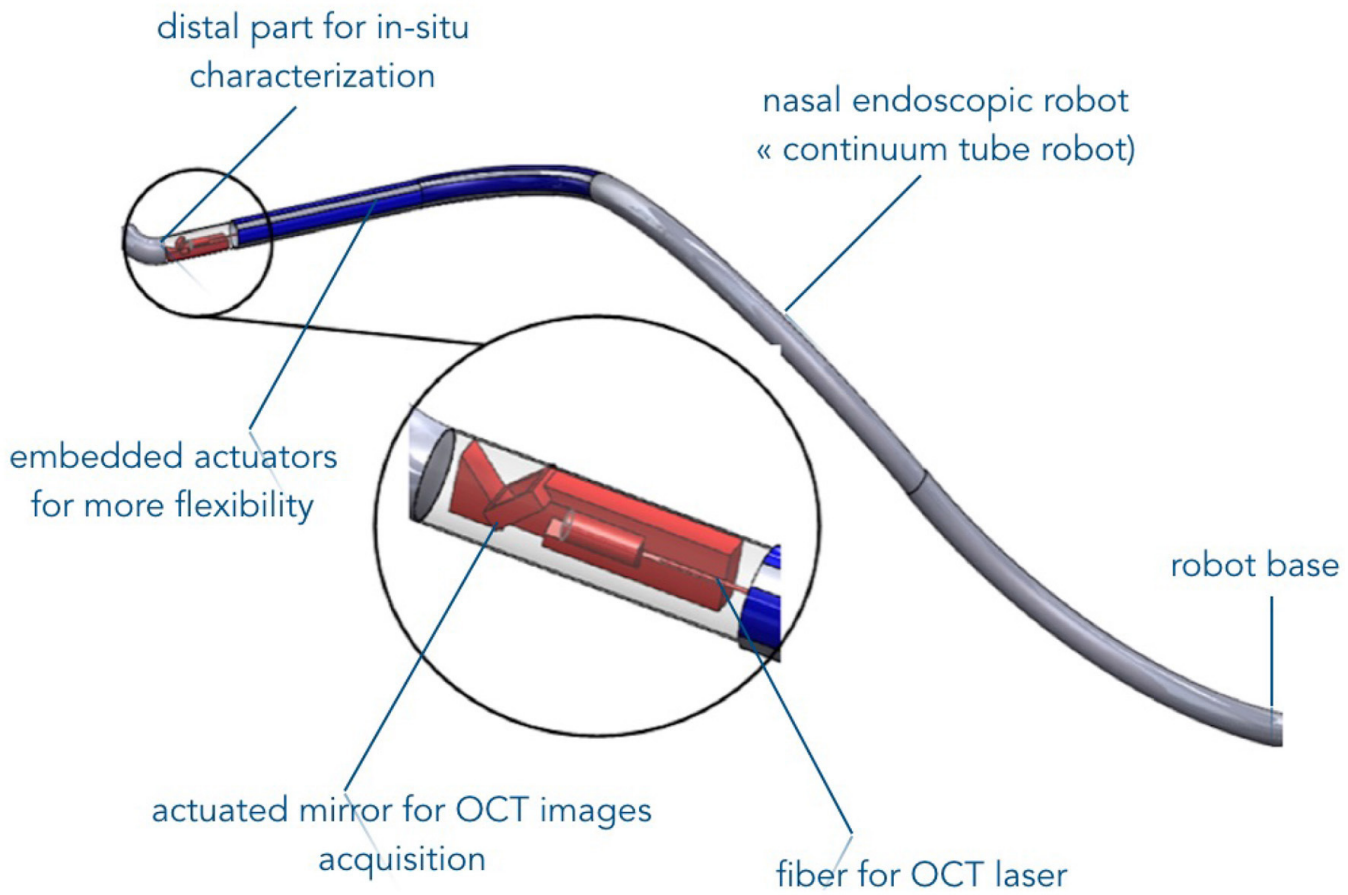
Illustration of the endonasal robotic concept under development for *in vivo* OE characterization (Chikhaoui et al., 2016).

Promising results were obtained using confocal microscopy technique. The latter outperforms the OCT since it can be used for visualization both the OE structural layered organization and axons bundles without the need of slicing or specific tissue labeling as usually performed in histology. The noninvasive manner of this technique and the fact that it did not require specific labeling of the tissue makes it a reliable candidate for *in vivo* investigation on patients. Millimeter confocal microscopy probe already exists on the market. Its future *in vivo* use could therefore be possible, for instance, when the microrobotic system under development within the NEMRO project will be finalized (Figure 7).

One of the major challenges of the nasal cavity endoscopy is the early, sensitive and specific diagnosis of neurodegenerative diseases such as Alzheimer's disease. Both OCT and confocal microscopy are not efficient in the visualization of other precursor signs such as Amyloid-β peptide deposits within the OE tissues. In our study, we investigated the potential of the well-established CellVizio system that requires fluorescence to detect the presence of Amyloid-β peptides deposit on OE tissues. One of the arguments in favor of the used of CellVizio probe is that it is available on different sizes ranging from a few hundred micrometers to a few millimeters that offer spatial resolution between 1.4 and 3.5 μm. In addition, depending on the considered probe, it is possible to visualize the tissue at different depths until 100 μm below the surface, i.e., able to observe independently different tissues layers.

Preliminary results have demonstrated that CellVizio technology allowed the visualization of the different elements of the OE tissues. This technology could enable the identification of the presence of Amyloid-β peptides deposits within the OE tissues. Indeed, we presumed that the green spots shown in Figure 6C could be Amyloid-β deposits given that the whole OE (not cut in 10 μm section) was stained by immersion with an primary antibody targeting Amyloid-β peptides. However, further developments and improvements are required to establish a more trivial and non-invasive tissues labeling procedure, particularly in case of *in vivo* investigation on patients. Actually, a conceivable method for *in vivo* labeling tissues on patients could be the delivery of fluorescent agent by spraying.

Besides Amyloid-β deposition, confocal microscopy technique demonstrated its ability to highlight potential structural changes and morphological alterations within the OE tissues or in retina as recently reported in the literature. Indeed, various studies in patients suffering from Alzheimer's disease as well as in animal models reported that retinal structural deficits such as peripapillary atrophy, thinning of the macular ganglion cell complex, axonal degeneration in the optic nerve, or cellular degeneration associated to visual dysfunctions (Hart et al., 2016). It can be hypothesized that these structural deficits of the retina that occur prior to the first signs of memory or motor loss in Alzheimer's patients, could be also identified within the OE tissues, starting with neurofibrillary tangles (Talamo et al., 1989) or axonal degeneration (Kovacs et al., 1999).

Future works will focus on reproducing the described methods and results on human tissues. First, we will start with the evaluation of the insertion and navigation of such imaging tools on human cadavers. To do this, it is necessary to integrate the imaging tools into the robotic endoscopic system for further *in vivo* characterization of OE tissues. A 2:1 prototype of the robotic endonasal system (Figure 7) is already developed and its functionalities are currently being tested on nasal phantoms. In the longer term, the developed robotic device system will serve as a safe intranasal navigation system without collisions with the nasal walls. If part of the OE is accessible in an almost straight line between the entrance of the nasal slots and the beginning of the OE, the rest of the tissues is unreachable. The flexible endonasal robot could address this concern. Additionally, the robot has a free internal channel that will allow inserting the characterization imaging system such as the CellVizio probe.

As a reminder, the objectives of this work was the evaluation of the capabilities of advanced imaging tools to highlight disorders that occur with the OE tissues. These disorders can be considered as early signs of a neurodegenerative disease, a correlation that has been widely reported in the literature over the last two decades. Today, Alzheimer disease can only be definitively diagnosed post-mortem thanks to a histopathological examination or in the case where the progress of the disease is very significant. Currently, the main diagnosis tools are the lumbar puncture and the scintigraphy. The study of the olfactory epithelium tissues can provide Alzheimer's disease diagnosis, if not at an early stage, it can be at least a means of establishing the disease (Godoy et al., 2019).

## Data Availability Statement

The datasets generated for this study are available on request to the corresponding author.

## Ethics Statement

The animal study was reviewed and approved by Franche-Comté University's Animal Care Committee (protocol number: 2015-002).

## Author Contributions

All authors listed have made a substantial, direct and intellectual contribution to the work, and approved it for publication.

## Conflict of Interest

The authors declare that the research was conducted in the absence of any commercial or financial relationships that could be construed as a potential conflict of interest.

## Footnotes

^1^Microrobotic nasal endoscopy by OCT: impact of smell deficiency on neurodegenerative diseases.

^2^www.jax.org

^3^www.janvier-labs.com/en

^4^www.nc3rs.org.uk/arrive-guidelines

^5^eur-lex.europa.eu/legal-content/EN/TXT/?uri=celex%3A32010L0063

^6^www.sigmaaldrich.com

^7^www.maunakeatech.com/en/cellvizio

^8^www.vivosight.com

^9^www.vivascope.de
